# Monetary incentives to avoid deforestation under the Reducing emissions from deforestation and degradation (REDD)+ climate change mitigation scheme in Tanzania

**DOI:** 10.1007/s11027-014-9607-y

**Published:** 2014-09-10

**Authors:** Meley M. Araya, Ole Hofstad

**Affiliations:** Department of Ecology and Natural Resource Management, Norwegian University of Life Sciences (NMBU), P.O. Box 5003, 1432 Ås, Norway

**Keywords:** Agricultural rent, Forest rent, Miombo woodland, Montane forest, Opportunity cost, REDD+, Sustainable harvest

## Abstract

The paper estimates and compares the level of Reducing Emissions from Deforestation and Degradation (REDD+) payments required to compensate for the opportunity costs (OCs) of stopping the conversion of montane forest and miombo woodlands into cropland in two agro-ecological zones in Morogoro Region in Tanzania. Data collected from 250 households were used for OC estimation. REDD+ payment was estimated as the net present value (NPV) of agricultural rent and forest rent during land clearing, minus net returns from sustainable wood harvest, divided by the corresponding reduction in carbon stock. The median compensation required to protect the current carbon stock in the two vegetation types ranged from USD 1 tCO_2_e^−1^ for the montane forest to USD 39 tCO_2_e^−1^ for the degraded miombo woodlands, of which up to 70 % and 16 %, respectively, were for compensating OCs from forest rent during land clearing. The figures were significantly higher when the cost of farmers’ own labor was not taken into account in NPV calculations. The results also highlighted that incentives in the form of sustainable harvests could offset up to 55 % of the total median OC to protect the montane forest and up to 45 % to protect the miombo woodlands, depending on the wage rates. The findings suggest that given the possible factors that can potentially affect estimates of REDD+ payments, avoiding deforestation of the montane forest would be feasible under the REDD+ scheme. However, implementation of the policy in villages around the miombo area would require very high compensation levels.

## Introduction

Conversion of forestland to agricultural land has been one of the main proximate causes of tropical deforestation (Barbier and Burgess 1997; Kaimowitz and Angelsen 1998; FAO 2010; Kissinger et al. 2012), and subsequent emissions of greenhouse gases and loss of other important ecosystem services (FAO 2010). This activity has been estimated to account for about 80 % of deforestation globally (Kissinger et al. 2012) and up to 12 % of total human-induced CO_2_ emissions (van der Werf et al. 2009). Moreover, deforestation due to agricultural expansion is expected to increase in the near future (Kissinger et al. 2012) despite the fact that, for example, tropical forests play an important role in regulating global climate by serving as sinks for carbon, storing about 50 % of the terrestrial organic carbon (FAO 2005). In the global climate change mitigation agenda, this led, in 2008, to the development of the United Nations’ policy measure Reducing Emissions from Deforestation and Forest Degradation (REDD), which in 2010 became REDD+ (Reducing Emissions from Deforestation and Degradation, and enhancing forest carbon stocks).

REDD was originally a financial-incentives-based policy measure developed as a means to protect forest carbon stocks from being released into the atmosphere as a result of deforestation and forest degradation (Wertz-Kanounnikoff and Kongphan-apirak 2009; Dudley 2010). REDD+ includes the possibility of offsetting carbon emissions through forest conservation, sustainable management of forests, and enhancement of forest carbon stocks (Bosetti and Rose 2011; Angelsen et al. 2012). Moreover, the policy measure provides a recognized system of payment for ecosystem services related to carbon sequestration and storage, whereby payments are made on the basis of performance (Wunder and Wertz-Kanounnikoff 2009).

REDD+ involves restrictions on local use of forestland and resources. However, about 1.6 billion people depend on tropical forests for their daily needs (World Bank 2004), and about 13 million ha of forest are cleared annually to provide livelihoods, incomes, and employment for millions of people in the tropics (FAO 2010; Kissinger et al. 2012). REDD+ will therefore impose considerable opportunity costs (OCs) on local communities (Wollenberg and Springate-Baginski 2009; Springate-Baginski and Wollenberg 2010). The OCs refer to the net benefits forgone by landowners/users as a result of not deforesting or degrading forests. The idea behind the REDD+ scheme is that such costs should be compensated in order to eliminate or reduce deforestation and forest degradation without affecting the livelihoods of local communities.

Several studies have shown that avoiding deforestation would cost less than USD 5 tCO_2_^−1^ (Osborne and Kiker 2005; Grieg-Gran et al. 2006; Stern 2007; Bellassen and Gitz 2008; Potvin et al. 2008; Yamamoto and Takeuchi 2012). Even though higher costs have been estimated (e.g., Kindermann et al. 2008; Butler et al. 2009), REDD+ is generally assumed to be a relatively cheap mechanism seen as a cost-effective climate change mitigation mechanism compared to industrial mitigation measures.

Most REDD+ cost estimates are based on payments required to offset OCs as the main cost component. The payments vary considerably among and within regions, depending on a number of ecological and economic factors (Angelsen 2008; Grieg-Gran 2008; Olsen and Bishop 2009). OCs of not converting forests to cropland vary greatly, depending on the types of farming systems and crops. For example, small-scale subsistence farming does not generally generate produce with quantifiable economic value and therefore the estimated OC of stopping such a farming system is usually very low, as shown by several studies (e.g., Bellassen and Gitz 2008; Olsen and Bishop 2009), compared to the cost of paying a landowner/user to not convert forests to large-scale commercial agriculture such as palm oil production (Butler et al. 2009) or soybean cultivation (Kindermann et al. 2008). Most OC calculations overlook the non-monetary benefits derived from agricultural and forestry activity that a landowner/user would forego by not clearing or degrading forests due to the difficulty in calculating them. However, some studies show that incorporating such costs into REDD+ projects increases the level of compensation (Karky and Skutsch 2010), implying that REDD+ compensation schemes may not be as cheap as many estimates suggest. Moreover, compensation values estimated for moist tropical forests with carbon densities of greater than 200 tC ha^−1^ in, for example, Brazil (Olsen and Bishop 2009), Cameroon (Bellassen and Gitz 2008), Guyana (Osborne and Kiker 2005), and Indonesia (Yamamoto and Takeuchi 2012) are very small, below USD 5 tCO_2_^−1^. However, such estimates cannot be treated as representative of dry tropical forests, such as those Tanzania in which carbon densities are less than 100 tC ha^−1^ (Munishi et al. 2010). Further, significant variations have been reported in the amount of compensation required to protect relatively intact forests, compared to degraded forests of the same type (Olsen and Bishop 2009). Other factors that affect the compensation estimates include variations in assumptions regarding the cost of labor (mainly family labor), the discount rate, time horizon, the degree of access to forests permitted after the implementation of the policy, and how net costs of conversion are treated (Grieg-Gran 2008; Karky and Skutsch 2010).

The aforementioned issues suggest the need for localized estimates of REDD+ payments and for identifying conditions and factors that will affect the feasibility of the policy measure and thereby inform decisions as to where to establish projects. To address these issues, we therefore conducted a study in Morogoro Region, Tanzania. Two vegetation types representing a humid forest and a dry tropical forest were considered. The main objective of this paper is to estimate and compare monetary incentives required to stop the conversion of montane forest and miombo woodlands into cropland under different cropping systems, and thus evaluate the feasibility of REDD+ policy in two agro-ecological zones in Tanzania. In addition, the effects of different assumptions regarding valuing farmers’ own labor in crop production as well as possible forest management rules that apply to forest use following the implementation of the scheme on the level of REDD+ payments required are examined to determine their implications for the policy design.

## Forest status in Tanzania

Tanzania possesses about 35 million ha of forests and woodlands, covering 40 % of the country’s total land area (FAO 2010). About 90 % of the forested and wooded area is made up of miombo woodlands (Malimbwi et al. 2005). Miombo, a collective name for species of the genera *Brachystegia*, *Julbernardia*, and *Isoberlinia*, is a common vegetation type in large parts of central, south, and eastern Africa (Campbell 1996). Other vegetation types include montane forests, mangrove forests, acacia savannah, and coastal forests (Burgess et al. 2010). About 52 % of the forests and woodlands in the country are within protected areas, and the remaining percentage is on village or general (*de facto* open access) lands (Zahabu et al. 2007; Mbwambo et al. 2012). Tanzania is one of a number of countries currently experiencing a high rate of deforestation and is planning to implement a REDD+ policy. It has been estimated that the country lost an average of 403,000 ha (about 1.02 %) of its forests and woodlands per year during the period 1990–2000 (FAO 2010). During the same period, the percentage loss was higher for miombo woodlands (13 %) compared to the Eastern African coastal forest mosaic (7 %), mangrove forest (2 %), and forests of the Eastern Arc Mountains (EAM) (1 %) (Burgess et al. 2010). Between 2000 and 2010, the annual forest loss increased to 1.1 % (FAO 2010). Agricultural expansion has been identified as one of the main drivers of deforestation in Tanzania (Makundi and Okiting’ati 1995; Angelsen et al. 1999; Burgess et al. 2002; Luoga et al. 2005; Kissinger et al. 2012). In addition, particularly the miombo woodlands have been subject to extensive extraction of wood for charcoal making (Monela et al. 1993; Luoga et al. 2005; Nduwamungu et al. 2008). It has been suggested that REDD+ monetary incentives may help to reduce the problem of deforestation and degradation in the Tanzania (Zahabu et al. 2007).

## Materials and methods

### Study sites

**Fig. 1:**
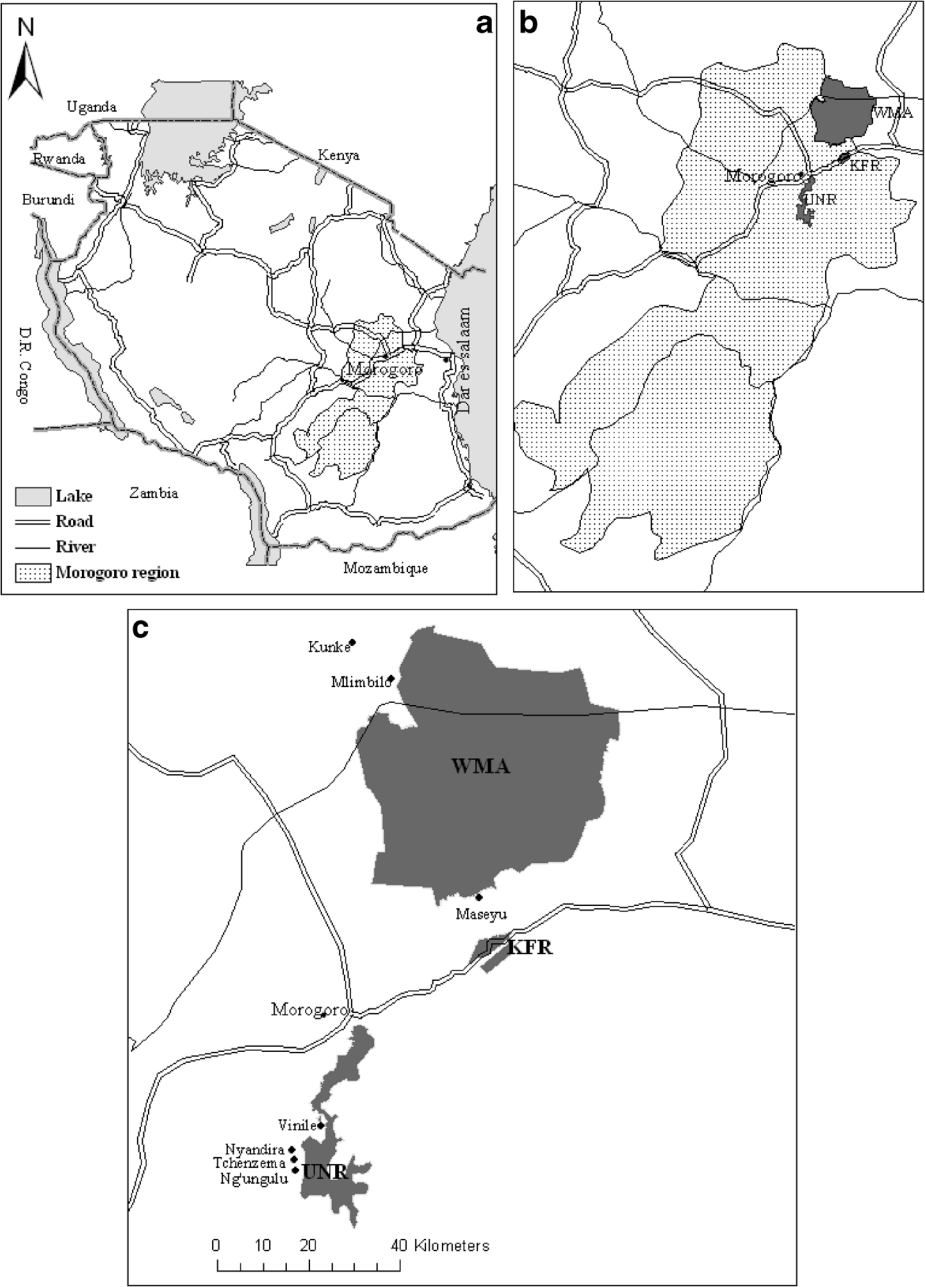
Tanzania (**a**), the location of Morogoro region (**b**), and the study villages and protected forests within the region (**c**)

Our socioeconomic survey was conducted in seven villages within Morogoro Region: Kunke, Maseyu, Mlimbilo, Ng’ungulu, Nyandira, Tchenzema, and Vinile (Fig. 1). The first three aforementioned villages are located at an average altitude of 400 m a.s.l., hereafter referred to as the lowland zone. The remaining four villages are located between 1,000 m a.s.l. and 2,668 m a.s.l., hereafter referred to as the highland zone. The two agro-ecological zones comprise two distinct vegetation types: miombo woodlands in the lowland zone and montane forest in the highland zone.

Kunke and Mlimbilo are located in Mtibwa Ward, 120 km north of Morogoro town. The villages cover an area of about 24,000 and 13,600 ha and have about 3,500 and 2,000 inhabitants, respectively. The climate of the area is sub-humid tropical, with a mean annual rainfall in the range of 800–1,200 mm. There are two rainy seasons, the short rains from October to December and the long rains from March to June. Mean annual temperature varies between 28 °C and 31 °C. The area includes settlements, a sugar factory, croplands, sugarcane (*Saccharum officinarum* L.) and teak (*Tectona grandis* L. leaf) plantations, grazing land, open scattered miombo woodlands, and part of a protected miombo woodland known as the Wami-Mbiki wild animals management area (WMA). The inhabitants depend mainly on agriculture for their livelihoods. They practice small-scale and medium-scale farming and cultivate crops for subsistence [maize (*Zea mays* L.)] and for marketing [rice (*Oryza glaberrima* S.), sesame (*Sesamum indicum* L.), and sugarcane]. WMA is a community-based conservation area that was established in 1999. The area is located in two regions, Morogoro Region and Tanga Region. It covers a core area of about 3,000 km^2^ and a buffer area of about 1,200 km^2^ (Madulu 2005). The WMA is surrounded by 24 villages including Kunke and Mlimbilo, and houses about 65,000 people in total; it covers 65 % of Kunke and 47 % of Mlimbilo. The woodlands inside the WMA have suffered from extensive tree cutting for charcoal production, and agricultural expansion and encroachment.

Maseyu is located in Gwata Ward, about 50 km northeast of Morogoro town and 150 km west of Dar es Salaam (Fig. 1). The climatic conditions and rainfall pattern in Gwata Ward are similar to those in Mtibwa Ward. The village of Maseyu covers approximately 36,000 ha and has about 2,000 inhabitants. It comprises settlements, croplands, open miombo woodlands, village reserves, part of Kitulangalo Forest Reserve (KFR), and a part of the WMA. About 80 % of the households depend on crop production (Nduwamungu et al. 2008). The local farming system is characterized by small-scale rain-fed crop cultivation for both subsistence consumption [maize, millet (*Eleusine coracana* L.), and sorghum (*Sorghum bicolor* L.)] and for marketing (sesame). The inhabitants are highly dependent on forest resources in protected and unprotected woodlands, both for their own consumption (firewood, fruit and vegetables, and wood for poles) and for commercial purposes (charcoal). The KFR spans two villages: Gwata and Maseyu. The reserve covers an area of about 435 ha in Gwata and 1,705 ha in Maseyu. The vegetation type is generally characterized as open, dry miombo that is dominated by the tree species *Julbernadia globiflora*, *Brachystegia boehmii*, and *Pterocarpus rotundifolius* (Luoga et al. 2000). The part of the forest reserve located in Maseyu is managed jointly by the central government and the village, while the part in Gwata is managed by the central government through Sokoine University of Agriculture in Morogoro town. The KFR was established in 1985 (Luoga et al. 2002). However, the current management systems have been practiced since 1995 in Gwata and since 2000 in Maseyu. The reserve is formally protected, but in practice, tree cutting is common practice and there have been some cases of conversion into agricultural land. An estimated 1.2 m^3^ ha^−1^ of wood is illegally harvested from the reserve annually (Luoga et al. 2002). Moreover, in the period 1975–2000, closed woodland in the reserve and the surrounding area declined by 45 %, while open woodland in the surrounding area increased by 42 % (Nduwamungu et al. 2008).

Ng’ungulu, Nyandira, Tchenzema, and Vinile are located in Mgeta Division, about 200 km west of Dar es Salaam (Fig. 1). Mgeta Division covers an area of 1,800 km^2^ and includes four wards and 28 villages with a total population of about 46,000. The climate in the area is tropical, with an average annual rainfall in the range of 1,000–2,000 mm. The rainy season lasts from November to May. The mean annual temperature varies from 15 °C to 21 °C (Kapilima 1994). The surrounding farmlands extend up to the border of Uluguru Nature Reserve (UNR), at about 2,000 m a.s.l., but rarely extend into the reserve (Frontier-Tanzania 2005). The inhabitants in areas to the UNR practice intensive small-scale farming of both subsistence crops [maize and pulses such as beans (*Phaseolus vulgaris* L.), chickpeas (*Cicer arietinum* L.), and peas (*Pisum sativum* L.)] and cash crops [vegetables such as cabbage (*Brassica oleracea* L.), potato (*Solanum tuberosum* L.), and tomato (*Solanum lycopersicum* L.)], and rely mainly on family members for labor. The UNR is part of the EAM, a chain of mountains stretching from the Taita Hills in southern Kenya through eastern Tanzania to the Udzungwa Mountains of south-central Tanzania. The nature reserve covers an area of approximately 24 km^2^ and is surrounded by 57 densely populated villages (FBD 2009). It consists of sub-montane forests (below 1,500 m a.s.l.), montane forests (1,600–2,400 m a.s.l), and upper montane forests (above 2,400 m a.s.l), and receives the highest rainfall (up to 4,000 mm year^−1^) in Tanzania (Lovett and Pócs 1993). Rivers originating in the reserve are the main supply of water to the local communities, as well as to Morogoro town and Dar es Salaam (Lundgren 1978). The UNR is one of the world’s biodiversity hotspots (Poynton et al. 2007). It also plays an important role as a carbon reservoir, currently storing about 10.3 million tons of carbon (FBD 2010). The reserve, which is managed by the central government, has been protected since the 1910s; in 1950, it was gazetted, and since then the cutting of trees has been officially prohibited. However, illegal harvesting of wood from the UNR has continued. Moreover, between 1955 and 2000, 25 % of the forest was lost, mainly due to agricultural expansion (Burgess et al. 2002). To mitigate the impact, the government allowed households in the surrounding villages to collect firewood, fruits, herbs, and other non-wood forest products for their own consumption, two times per week (FBD 2009). Uluguru Nature Reserve comprises four separate forest reserves, Uluguru North FR, Uluguru South FR, Bunduki FR, and Bunduki Gap FR. Our study was conducted in Mgeta Division, which covers the southwestern side of the Uluguru Mountains, within Uluguru South FR.

### Data

The data were collected over the course of 5 months, between September 2011 and January 2012, from communities located in the seven study villages. A total of 112 households were randomly selected from the four villages in Mgeta Division (Ng’ungulu, Nyandira, Tchenzema, and Vinile), 65 from the two villages in Mtibwa Ward (Kunke and Mlimbilo), and 54 from the village of Maseyu in Gwata Ward. Basic demographic information, as well as information relating to costs and revenues associated with crop production and forestry, local prices of all inputs and outputs, as well as labor costs, was obtained using structured questionnaires. Further information was collected through focus group discussions to triangulate and verify the information obtained from households as well as to obtain qualitative information. In addition, we visited local market places to gather information on prices of inputs and outputs. Moreover, secondary sources were used to obtain data on the carbon storage potential of different types of vegetation and land uses.

#### Estimating REDD+ payments to offset OCs

The OC of forest protection can be defined as the forgone net benefits from the best alternative land uses (Bond et al. 2009; Olsen and Bishop 2009; Angelsen et al. 2012) and are payments required to compensate forest owners or users for their forgone benefits (Angelsen 2008). REDD+ payments to offset OCs can be estimated as the net present value (NPV) of the next best use of forestland divided by the associated reduction in carbon stock (Wertz-Kanounnikoff 2008). OC estimates will depend on the rules that apply to forest use after the REDD+ contract has been signed. For simplicity, we assumed that deforestation should stop immediately, while wood harvesting could continue in a sustainable way. Here we defined sustainable harvest as the mean annual increment (MAI) of the forest or woodland under consideration. We therefore estimated the REDD+ payments to offset the OCs of stopping conversion of the two vegetation types considered in our study as the NPV of agricultural rent and forest rent during land clearing, minus the net returns from sustainable wood harvest, divided by the corresponding possible reduction in carbon stock.

#### Estimating agricultural rent

Data on crop production were collected from 606 sample plots. The size of the sample plots ranged from 0.1 to 2.0 ha in Mgeta Division, 0.4 to 5.0 ha in Mtibwa Ward, and 0.4 to 6.0 ha in Gwata Ward. The percentage share of each crop type cultivated in each village is presented in Table 1. We estimated the financial returns from the major crops grown at each village. The costs of crop production included the cost of land clearing, seeds, fertilizers, pesticides and fungicides, labor required for different activities, and transportation to the local market, and we obtained from local markets the prices of each input and each type of crop produce. The median yield and average price of the major crops cultivated in each village are summarized in Tables 2 and 3, respectively. Due to the difficulty of valuing a farmer’s own labor (Fisher et al. 2005; Le 2009), we applied and compared three different wage rates: (1) the reported wage rate in the study villages (hereafter referred to as the “village wage rate”), (2) the minimum wage rate for agricultural labor in Tanzania (“minimum wage rate”), and (3) a wage rate equal to zero (“zero wage rate”) which assumed an opportunity cost of labor as zero. The net benefit (NB) of each crop type was calculated as the difference between the total value of the crop harvest and the value of all production factors except land. Assuming that present agricultural practices are sustainable and that the relative prices of products and factors are constant, the NPV of land can be calculated as annual NBs divided by a constant discount rate. However, the assumption that the current yield estimates can reflect past and future yield values might lead to underestimates or overestimates of past and future estimates, respectively, as the croplands considered in our study were not representative of recently deforested lands. Further, the choice of the appropriate discount rate is far from an obvious and straightforward decision (Howarth and Norgaard. 1992). We chose a nominal discount rate based on the rate of lending by the Bank of Tanzania as of January 2011 (12 %) (BOT 2011). The real interest rate (*r*) was estimated by adjusting the nominal discount rate for inflation using the Fisher equation

**Table 1:** Percentage share of crops cultivated in each study village

Village	Crop type (% share)									
	Maize	Sugarcane	Rice	Maize and sesame	Maize and millet	Millet and sesame	Vegetables	Pulses	Maize and pulses	Maize and vegetables
Kunke	50	13	16	21						
Maseyu	49			18	18	15				
Mlimbilo	76	6	14	4						
Ng’ungulu	19						1.5	4	74	1.5
Nyandira	8.5						13	6	50	22.5
Tchenzema	11						4	10	47	28
Vinile	13								74	13

**Table 2:** Median harvest (ton ha^−1^) of the major crops cultivated in each study village

Crop type	Village						
	Kunke	Maseyu	Mlimbilo	Ng’ungulu	Nyandira	Tchenzema	Vinile
Maize	1.24	0.62	0.99	0.49	1.78	0.67	0.20
Sugarcane	19.77		49.00				
Sesame	0.50	0.30	0.86				
Rice	0.88		0.53				
Vegetables				0.49	1.73	3.08	
Pulses				0.49	0.37	0.30	0.43

**Table 3:** Average price (USD kg^−1^) of the major crops cultivated in each study village

Village	Price (USD kg^−1^)					
	Maize	Sugarcane	Sesame	Rice	Vegetables	Pulses
Kunke	0.22	0.17	0.82	0.91		
Maseyu	0.25		0.64			
Mlimbilo	0.22	0.02	0.48	0.93		
Ng’ungulu	0.34				0.29	0.27
Nyandira	0.26				0.16	0.47
Tchenzema	0.29				0.14	0.39
Vinile	0.27					0.38

$$ r=\frac{1+i}{1+\pi }-1 $$

Where *i* is the nominal interest rate and π is the inflation rate. The inflation rate of all items for the period January 2010 to January 2011 was 6.4 % (BOT 2011). Accordingly, we used a discount rate of 5.3 %. The median agricultural NPV per hectare in each village was obtained using the formula

$$ \mathrm{NPV}=\mathrm{FR}+{\alpha}_{\mathrm{i}}\left({\mathrm{NB}}_{\mathrm{i}}/r\right)+\dots {\alpha}_{\mathrm{n}}\left(\mathrm{NBn}/r\right) $$

where FR is the net revenue obtained per hectare from forest clearing, NB_*i*_ represents the median NBs per hectare from the *i*th most cultivated crop in each village, *α* ∈ [0, 1] is the ratio of cultivation of *i*th crop per hectare, and *r* is the real discount rate.

#### Estimating forest rent

##### Montane forest

The montane forests are extensively used for harvesting wood for tool handles, poles, and timber. The tree species used for these products were identified and their net profits were estimated. Timber-producing species account for approximately 25 % of the total average standing volume (319 m^3^ ha^−1^). Tree species important for making poles and tool handles accounted for respectively 11 % and 16 % of the total average tree density (539 stems ha^−1^). Timber volume was estimated using 63 % log recovery and 30 % timber recovery (Muthike et al. 2013). In contrast to the production of agricultural produce in the studied villages, the production of logs and timber requires labor that is more skilled. Hence, it is assumed that labor is hired for these activities. Wood extracted for making the different products is considered to be a free good and thus is available free of cost to the producer.

The collection of firewood from the reserve is assumed to be less or equal to the MAI of the forest, and therefore sustainable harvesting means only the collection of firewood. A household collects two head loads of firewood per week, and an average weight of a head load is about 13 kg (Mitinje et al. 2007). About 16,000 households depend on the nature reserve for firewood. The total biomass of firewood collected per hectare was therefore estimated as the number of households multiplied by the average annual consumption of a household divided by the total area of the forestland. The NPV was then calculated by dividing the value by the real interest rate, which was 5.3 %. The economic parameters that we used to estimate rents to firewood, poles, timber, and tool handles are presented in Table 4.

**Table 4:** Economic parameters used to estimate rents to firewood, poles, timber, and tool handles

Product type	Quantity per hectare	Unit	Labor cost per unit (USD)	Price per unit (USD)
Firewood	0.90	Ton	0	106.00
Poles	170	Each	1.62	12.72
Timber	15.32	m^3^	39.60	56.71
Tool handles	513	Each	0.32	0.64

##### Miombo woodlands

Most of the information on the miombo woodlands was derived from the data we collected from the village of Maseyu (Gwata Ward), and we assumed that the collected data were also representative of similar miombo woodlands in Mtibwa Ward. The average standing volume on public land (degraded miombo) is 14 m^3^ ha^−1^ (Zahabu 2008). We estimated that the average standing volume in the forest reserve (dense miombo) in 2011 was 65 m^3^ ha^−1^, on the basis of inventory data that was collected in the same year. The MAI of the woodlands around the study villages is estimated to be 2.9 Mg ha^−1^ for regrowth miombo and 3.7 Mg ha^−1^ for old-growth miombo (Ek 1994), equivalent to a volume of 3.41 m^3^ ha^−1^ and 4.35 m^3^ ha^−1^, respectively, using a 0.85 conversion factor from biomass (ton) to volume (m^3^) (Malimbiwi et al. 1994). Tree species used for charcoal making represent 40 % of the standing volume. One cubic meter of wood yields 4.3 bags of charcoal (56 kg/bag), and the labor required to produce one bag of charcoal is 2.3 person-days (Hofstad 1997; Luoga et al. 2002). The cost of physical inputs, such as axes, machetes, and rope, is approximately USD 6.4. Such inputs are assumed to last for 5 years. We found that the average price of one bag of charcoal ranged from USD 5 at the kiln site to USD 6.3 at the road side. The same assumption as for the agricultural rent was made for estimating cost of labor required for different activities such as felling and cross-cutting of trees, log piling, stacking, and loading and unloading charcoal kilns. The same assumption as for the montane forest products was made about value of wood extracted for charcoal production.

#### Estimating carbon stocks in forests, woodlands, and agricultural lands

Data on carbon stocks in different pools of the two studied forest types (miombo and montane forest) and surrounding agricultural lands were obtained from various published sources (Table 5). Averages were taken when more than one estimate per vegetation type was available. A rough estimation of biomass was also made in the WMB area and the surrounding open woodland for comparison with the biomass data obtained from other miombo woodlands. Ten plots were sampled using an inventory design proposed by the National Forestry Resources Monitoring and Inventory of Tanzania and similar biomass densities were found in WMB as reported for miombo (Luoga et al. 2002). The below-ground biomass of miombo woodlands was estimated as 20 % of the above-ground biomass. The soil carbon of croplands in original miombo woodlands was estimated at 60 % of soil carbon in miombo woodlands (Walker and Desanker 2004). The carbon estimate was multiplied by the conversion factor of 3.67 to obtain tons of carbon dioxide equivalents (tCO_2_). The net carbon or equivalent carbon dioxide that will be protected under the REDD+ scheme was estimated as the mean of the total carbon storage of each vegetation type minus the mean of the total carbon storage under the corresponding alternative land use (i.e., agriculture).

**Table 5:** Distribution of carbon stock (Mg ha^−1^) by carbon pool in different land-use categories in the lowland and highland agro-ecological zones in Tanzania

Agro-ecological zone	Land-use type	Above-ground biomass	Dead wood	Litter	Below-ground biomass	Soil	Total	Source^a^
Highland	Afro-montane forest (a)	222.0	11.0	13.0	54.0	295.0	595.0	(2), (7)
	Crop land (b)	3.3	0.1	0.3	0.9	123.0	127.6	(7)
	Net loss (a-b)						467.4	
Lowland	Dense miombo woodland (a)	20.0	–	–	4.0	78.5	102.5	(1), (3), (4), (5), (6)
	Degraded miombo woodland (b)	3.5			0.7	78.5	82.7	(4), (8)
	Cropland (c)	0.6				47.1	47.7	(6)
	Net loss (a–c)						54.8	
	Net loss (b–c)						35.0	

^a^Sources: (1) Chamshama et al. 2004; (2) Munishi and Shear 2004; (3) Munishi et al. 2010; (4) Ryan et al. 2011; (5) Shirima et al. 2011; (6) Walker and Desanker 2004; (7) Willcock et al. 2012; (8) Zahabu 2008

#### Data analysis

Of the 606 sample plots, 593 were used for analysis after the data were cleaned. The remaining plots were omitted from the analysis due to incomplete information (e.g., because crops had not been harvested). To avoid the problem of non-normality caused by the fact that profitability on many plots was low while profitability on a few plots was very high, a Kruskal–Wallis test was used to detect statistically significant differences in the median NPVs of crop production as well as the median compensation estimates between the two agro-ecological zones and among the study villages within the zones. Non-parametric multiple pair-wise comparisons between different parameters were made using Tukey’s test and *t* tests, with a significance level of *α* = 0.05. The statistical software R version 3.0.1 (R Core Team 2013) was used for analysis and SigmaPlot version 11 (Systat Software Inc. 2008) was used for plotting.

#### Sensitivity analysis and elasticity estimation

The analysis in the base case scenario assumed a constant value for each parameter (crop yield, carbon density, prices of the different crop types, and discount rates). However, these variables may have changed over time, thus affecting the results of the analysis. Therefore, to assess the sensitivity of the base case results to changes in the most important parameters, a sensitivity analysis was conducted by making changes in key variables. Changes in discount rate and carbon stock value were considered in the analysis. Based on an assumption that the current discount rate (5.3 %) was quite low, we examined the effect of increasing the discount rate to 10 %. For the carbon stock, reductions and increases of up to 50 % were assumed. Changes in carbon stocks can be caused by fire, uncertainties in carbon stock estimates (Pelletier et al. 2012), or enhanced growth due to different forest management interventions. Moreover, we obtained the elasticity of results to changes in crop yield from an estimated production function.

## Results

### Agricultural rent

**Fig. 2:**
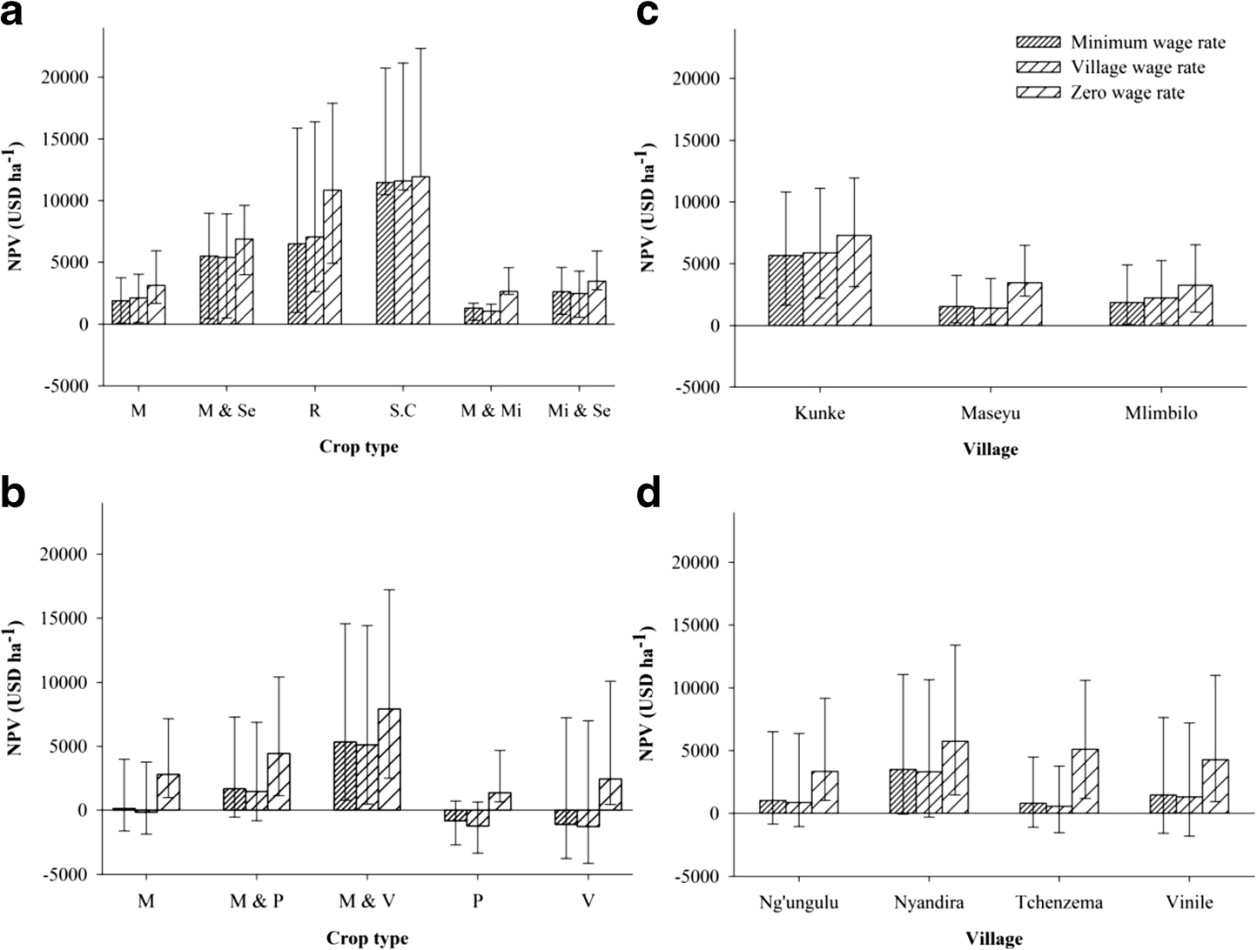
The median NPV (USD ha^−1^) of crop production by crop type (**a**, **b**), village (**c**, **d**) from the lowland zone (**a**, **c**), and the highland zone (**b**, **d**); the *lower* and *upper error bars* represent the first and third quartiles, respectively. *M* maize, *Mi* millet, *P* pulses, *R* rice, *S.C* sugarcane, *Se* sesame, *V* vegetables

**Fig. 3:**
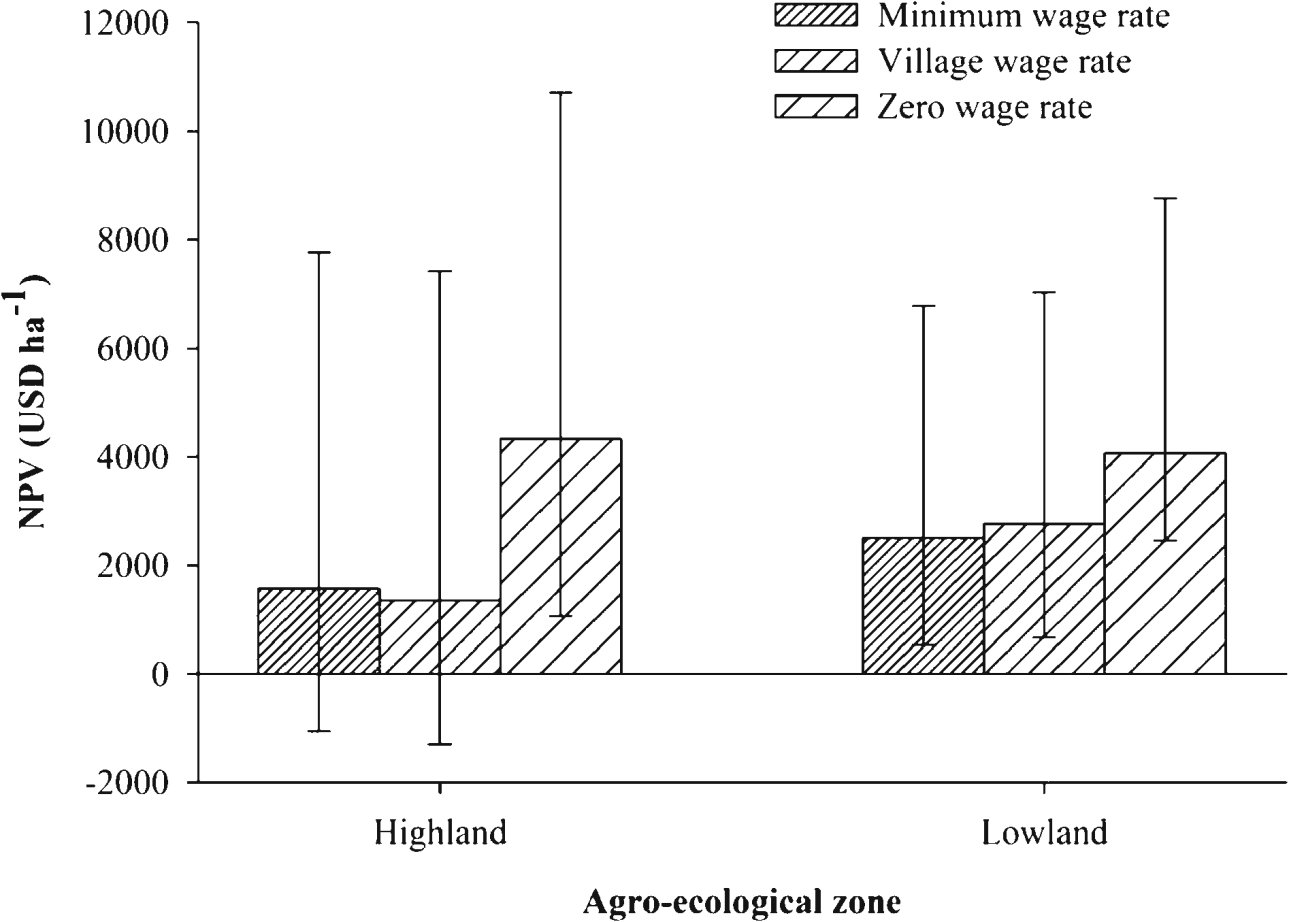
The median NPV (USD ha^−1^) of crop production in the highland and lowland zones; the *lower* and *upper error bars* represent first and third quartiles, respectively

Figure 2 shows the interquartile range (IQR) of the NPV estimates of each crop type across each study village and each agro-ecological zone. The median NPV estimates varied greatly across crop types, regardless of the wage rates applied. Maize cultivated with vegetables yielded a significantly higher (*p* < 0.001) median NPV in the highland villages, while sugarcane and rice yielded a significantly higher (*p* < 0.001) median NPV in the lowland villages. Depending on the wage rates used, the median NPV estimates also showed variation between the two agro-ecological zones; when the positive wage rates were considered, the median NPV of all the major crops was significantly higher in the lowland zone compared to in the highland zone. However, when the zero wage rate was considered, the median NPV tended to be higher in the highland zone, albeit not significantly (Fig. 3). Similarly, a significant variation in median NPV values between the study villages in the highland zone was observed when only one of the positive wage rates was used (Table 6). However, in the lowland zone, the median NPV between the villages showed significant variation regardless of the wage rates applied (Table 6). The results of the non-parametric multiple comparisons also confirmed variations in the median NPV between villages within the same agro-ecological zone. In the highland zone, when either of the positive wage rates was used, the median NPV estimate for Nyandira was significantly higher compared to the other three villages. However, differences in the median NPV value between the three villages, Ng’ungulu, Tchenzema, and Vinile, were insignificant. When the zero wage rate was considered, the median NPV estimate did not show significant differences among the villages. In the lowland zone, regardless of wage rate used, the median NPV estimate was significantly higher for Kunke compared to for Maseyu and Mlimbilo. However, the variation in the median NPV estimate for Maseyu and Mlimbilo was not significant. The difference in the NPV values (USD ha^−1^) for the same crop types was also very high, with the IQR ranging between 884 for maize cultivated with millet and 14,380 for rice in the lowland zone, and between 3,207 and 13,324, respectively, for pulses and maize cultivated with vegetables in the highland zone. When the zero wage rate was considered, the IQR values showed a slight increase.

**Table 6:** Variations in the median NPV of crop production between the study villages within the two agro-ecological zones

Agro-ecological zone	Minimum wage rate		Village wage rate		Zero wage rate	
	Kruskal–Wallis *χ* ^2^	*p* value	Kruskal–Wallis *χ* ^2^	*p* value	Kruskal–Wallis *χ* ^2^	*p* value
Lowland	15.17	<0.001	21.24	<0.001	16.44	<0.001
Highland	9.45	0.02	10.66	0.01	4.58	0.2

The different wage rates resulted in significantly different median NPV estimates for the major crops in all villages, except for Kunke (Table 7). Further, non-parametric multiple comparisons showed that the median NPV values estimated using a zero wage rate were significantly higher compared to estimates using both minimum and village wage rates. However, the minimum and village wage rates yielded similar median NPV estimates in both agro-ecological zones.

**Table 7:** Variations in the median NPV of crop production estimated using the different wage rates in each study village

Agro-ecological zone	Village	Kruskal–Wallis *χ* ^2^	*p* value
Lowland	Kunke	4.22	0.12
	Maseyu	18.18	<0.001
	Mlimbilo	5.73	0.049
Highland	Ng’ungulu	24.95	<0.001
	Nyandira	8.29	0.015
	Tchenzema	20.86	<0.001
	Vinile	20.74	<0.001

### Forest rent

In the montane forest, the net returns per hectare from the different forest products during forest clearing was estimated to be USD 1,887 from poles, USD 262 from timber, and USD 160 from tool handles. The NPV of firewood collection was estimated as USD 1,797 ha^−1^. The returns from the sustainable harvest were significantly higher compared to the returns from the other forest products during forest conversion. Table 8 lists the net returns from charcoal burning both during clearing and from annual sustainable harvest in the miombo woodlands. The results shows that regardless of the wage rates used, NPV estimates of annual sustainable charcoal production for both the forest reserve and the public land resulted in a relatively higher value compared to the return from charcoal production during conversion. With regard to the wage rates, as in the case of crop production, the NPV values estimated by using the zero wage rate were found to be significantly higher than those estimated using the two other wage rates.

**Table 8:** NPV of charcoal production during forest conversion and through annual sustainable harvest

Legal status	Activity	NPV (USD ha^−1^)		
		Minimum wage rate	Village wage rate	Zero wage rate
Public woodland	Clearing	52	82	161
	Annual sustainable harvest	554	856	1,644
Forest reserve	Clearing	262	400	763
	Annual sustainable harvest	715	1,100	2,106

### The OCs of stopping conversion of forestland to cropland

The net median income (USD ha^−1^) that would be lost from not converting forestland into cropland was in the range of USD 1,289–6,277, depending on the wage rates considered (Table 9). With regard to village wage rate, both the lowest estimates (USD 2,289) and the highest (USD 5,006) estimates were observed in the lowland zone. However, when zero wage rate was used, the lowest estimate (USD 2,719) was observed in the lowland while the highest (USD 6,277) was in the highland (Table 9). The return from forest products during land clearing accounted for up to 16 % of the total median OC in the lowland zone and 70 % in the highland zone, depending on the wage rates considered and the biomass density of the miombo woodlands. When the village wage rate was used, the OCs from forest rent during forest conversion represented 64 % in the highland zone and 10 % in the lowland zone. The results also indicated that incentives from sustainable annual wood harvest could offset up to 45 % of the total median OCs of protecting the miombo woodlands in forest reserves and up to 40 % for the miombo woodlands on public land, depending on the wage rates used. Similarly, the sustainable collection of firewood in the highland could offset about 55 % of the estimated total median OC with regard to the positive opportunity cost of labor.

**Table 9:** Opportunity costs of stopping conversion of forestland into cropland and the required REDD+ payments to offset those costs

Vegetation type	Village	NPV (OC) (USD ha^−1^) *r* = 5.3 %			REDD+ payment (USD tCO_2_) *r* = 5.3 %		
		Minimum wage rate	Village wage rate	Zero wage rate	Minimum wage rate	Village wage rate	Zero wage rate
Dense miombo	Kunke	4,916	5,006	5,631	25	25	28
	Maseyu	1,824	1,363	2,860	9	7	14
	Mlimbilo	2,452	2,633	3,177	12	13	16
Degraded miombo	Kunke	4,866	4,932	5,490	38	39	43
	Maseyu	1,775	1,289	2,719	14	10	21
	Mlimbilo	2,402	2,559	3,036	19	20	24
Montane forest	Ng’ungulu	1,823	1,580	3,991	1	1	2
	Nyandira	4,791	4,660	6,277	3	3	4
	Tchenzema	1,965	1,482	5,397	1	1	3
	Vinile	2,287	2,067	5,156	1	1	3

### REDD+ payments to offset OCs

**Fig. 4:**
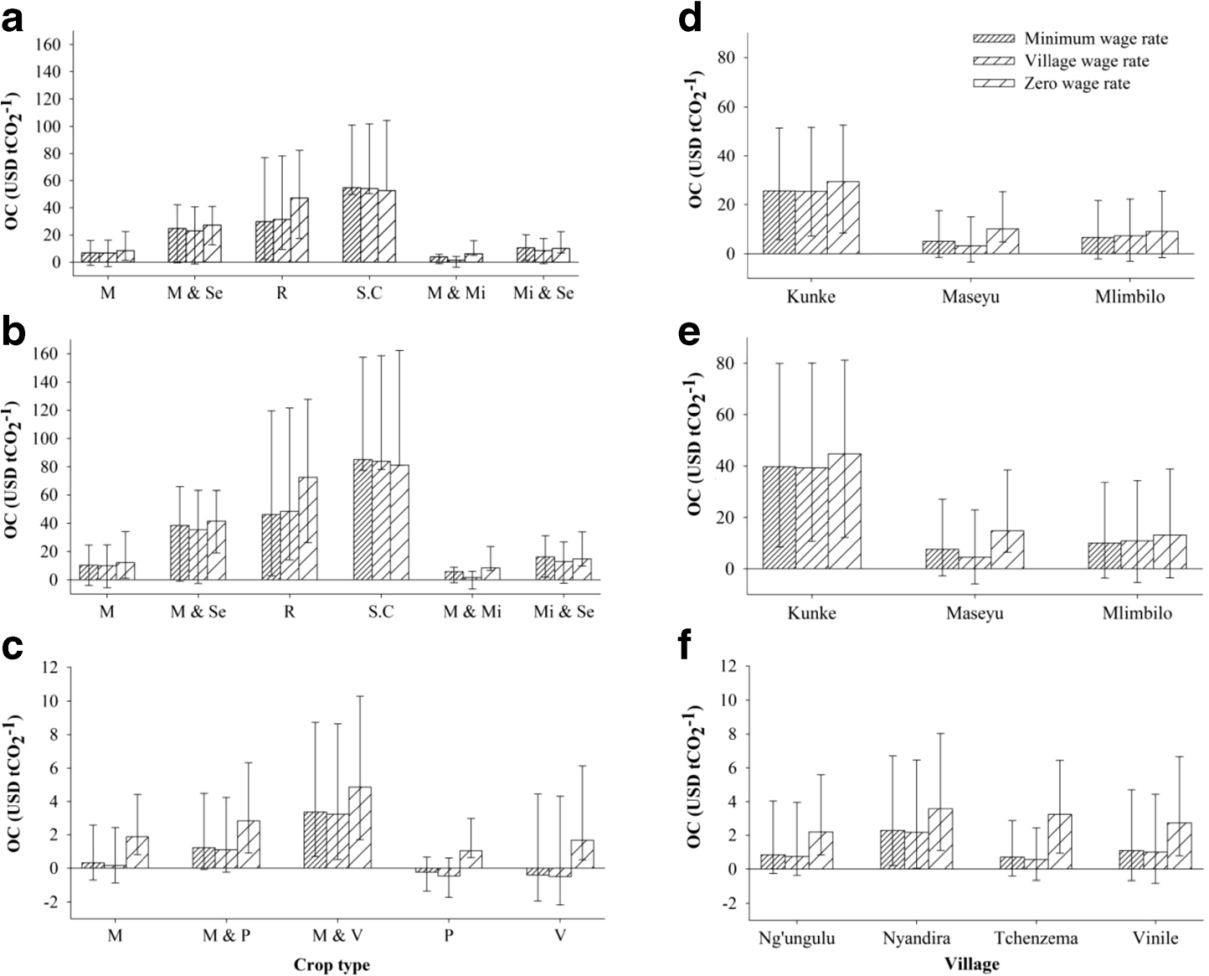
The median compensation (USD tCO_2_^−1^) by crop type (**a**, **b**, **c**) and village (**d**, **e**, **f**) in the lowland (dense miombo) (*upper panel*), lowland (degraded miombo) (*middle panel*), and highland (*lower panel*); the *lower* and *upper error bars* represent first and third quartiles, respectively. *M* maize, *Mi* millet, *P* pulses, *R* rice, *S.C* sugarcane, *Se* sesame, *V* vegetables

The level of compensation required to reduce emissions of CO_2_ by stopping the conversion of forestland to cropland differed significantly (*p* < 0.001) in the two agro-ecological zones, and hence also for the different vegetation types, regardless of the wage rates used (Fig. 4); it was significantly higher in the study villages in the lowland zone. With regard to the village wage rate, the median compensation required to protect the current carbon stocks in the different vegetation types varied from USD 1 tCO_2_^−1^ for the montane forest to USD 39 tCO_2_^−1^ for the miombo on public land (Table 9). The zero OC of labor increased the median compensation value to USD 3 tCO_2_^−1^ in the highland zone and to USD 43 in the lowland (Table 9). Regardless of the wage rate used, the median compensation estimates were also found to vary between villages within the same agro-ecological zones. When the village wage rate was considered, the median compensation ranged from USD 1 tCO_2_^−1^ for Ng’ungulu, Tchenzema, and Vinile to USD 3 tCO_2_^−1^ for Nyandira in the highland zone, and from USD 7 tCO_2_^−1^ for Maseyu to USD 39 tCO_2_^−1^ for Kunke in the lowland zone for the miombo woodlands on public land (Table 9). With regard to the zero wage rate, compensation estimates increased significantly, except for Kunke. With regard to the zero wage rate, when the compensation levels were calculated for each major crop separately, the variation in median compensation was high, ranging from USD 1 tCO_2_^−1^ for crops cultivated in the highland zone to USD 81 tCO_2_^−1^ for crops cultivated on formerly degraded miombo woodland in the lowland zone (Fig. 4). The OCs of avoiding cultivation of the most profitable crops after deforesting the relatively dense miombo woodlands were significantly higher compared to after deforesting the degraded miombo woodlands (Fig. 4).

### Sensitivity analysis

When village wage rate is considered, an increase in discount rate from 5.3 % to 10 % can reduce the median compensation values by about 50 % in the lowland and by up to 30 % in the highland zone (Table 10). Similarly, a 10 % increase and reduction in carbon density can result in a 9 % increase and an 11 % reduction in the median compensation estimates, respectively. Moreover, the median compensations would increase by 100 % for a 50 % reduction in carbon density and decrease by 33 % for a 50 % increase in carbon density. The midpoint yield elasticity (0.274) of agricultural rent obtained from the production function showed that the results are not sensitive to changes in yield.

**Table 10:** Results of the sensitivity analysis of REDD+ payments (USD tCO_2_^−1^)

Vegetation type	Village	REDD+ payment (USD tCO_2_^−1^)									
		Discount rate (*r*)		Carbon density (C)							
		*r* = 10 %		+10 %		−10 %		+50 %		−50 %	
		Village wage rate	Zero wage rate	Village wage rate	Zero wage rate	Village wage rate	Zero wage rate	Village wage rate	Zero wage rate	Village wage rate	Zero wage rate
Dense miombo	Kunke	14	17	23	26	28	31	17	19	50	56
	Maseyu	4	9	6	13	8	16	5	10	14	29
	Mlimbilo	8	10	12	14	15	18	9	11	26	32
Degraded miombo	Kunke	20	23	35	39	43	48	26	29	77	86
	Maseyu	5	12	9	19	11	24	7	14	20	42
	Mlimbilo	11	13	18	22	22	26	13	16	40	47
Montane forest	Ng’ungulu	1	2	1	2	1	3	1	2	2	5
	Nyandira	2	3	2	3	3	4	2	2	5	7
	Tchenzema	1	2	1	3	1	3	1	2	2	6
	Vinile	1	2	1	3	1	3	1	2	2	6

## Discussion: implications for REDD+ implementation design

### Agricultural rent

Variations in the median NPV were clearly related to crop types in both agro-ecological zones. We observed relatively higher median NPV estimates for villages with the highest percentage share of the most profitable crops. The reasons for choosing a certain crop type included agro-ecological considerations, distance to the nearest market, and farmland size. In Kunke, for example, sugarcane was the most profitable crop type cultivated on farmlands located close to the sugar factory; farmers able to grow sugarcane on 5 ha of land or more were able to sell their produce to the sugar factory. By contrast, rice, one of the most profitable crop types, is cultivated only in places with enough water, including Kunke and Mlimbilo but not Maseyu. In the highland zone, the continuous availability of water allows the local communities to cultivate vegetables (the most profitable crops in the highland villages) all year round. However, in villages located farthest from the local market in Nyandira, such as Ng’ungulu, the cultivation of vegetables is limited.

Our analysis of agricultural rent showed a high variation in and a skewed distribution of NPV. The majority of farmers earned little income from the cultivation of crops, and only a few were able to make high profits. Hence, for a given REDD+ payment, normally a few families would still find it more profitable to continue to practice deforestation rather than accept the compensation and stop expanding their croplands. Few buyers would find it profitable to offer a sufficiently high price to convince the most efficient farmers to stop deforestation.

Labor input is an important component of crop production, and it can be broadly categorized into family labor and hired labor. Labor was abundant in the study villages, and the main source was family labor, particularly in the highland zone. Family labor accounted for more than 90 % of the total labor used in crop production in the highland zone and 70 % in the lowland zone. With the exception of Kunke, significant variation was found in all study villages in the median NPV of crop production between those estimated with regard to the positive OC of labor and those estimated using the zero OC of labor. The variation was higher in three villages in the highland zone because the main activities, such as land preparation, were undertaken using family labor. For some farm tasks that might require more labor, such as weeding, labor exchanges among relatives and neighbors were practiced. By contrast, in the lowland zone, particularly in Kunke and in some parts of Mlimbilo, some of the major tasks were carried out by hired labor due to suitability of the area for using machinery such as tractors. The sugar factory took responsibility for harvesting on the sugarcane plantation. Our results show the significance of crop production in terms of providing employment opportunities for the villagers. The difference between the NPV values estimated using a zero labor cost and those estimated using a positive labor cost can be seen as foregone benefits in terms of lost employment opportunities. In places such as the areas in which the study villages were located, where there are no readily available alternative sources of income, forgone employment opportunities need to be considered when a REDD+ policy is designed. The availability of family labor together with the profitability of crop production, particularly in the lowland zone, implies that the villagers have possibilities and incentives for increasing their farm size wherever land is suitable. Further intensification of their high-value crops implies greater increases in the costs of implementing the REDD+ policy measure.

### Forest rent

The results showed that sustainable wood harvests from the montane forest and from both dense and degraded miombo woodlands resulted in a significantly higher NPV compared to the net returns from wood harvest during conversion to croplands. This was due to the relatively low discount rate used in the NPV estimation.

### The OC of stopping conversion of forestland to cropland

The income that would be lost during conversion is significant. The OC originating from wood harvest during land clearing was significantly higher in the highland zone, but it was offset by the relatively higher return from sustainable harvest in the zone. In the case of miombo woodlands, no difference in OC estimates was observed between the woodlands in the forest reserve and those on public land. The return from charcoal production during forest conversion was higher from miombo woodlands in the forest reserve compared to miombo woodlands on public land, mainly due to the relatively higher biomass density in the former case. However, since the return from sustainable harvest was also higher from miombo woodlands in the forest reserve than on public land, the OC was offset. The results also highlighted the significance of sustainable harvests in offsetting some of the OCs, depending on the biomass density of the vegetation. In places, such as the areas occupied by the study villages, where more than 90 % of the local people depend on biomass energy, such incentives might be more realistic and attractive to local communities than monetary incentives (Mohammed 2011). According to Kaczan et al. (2013), compared to cash payments, non-monetary payments in the form of fertilizers would motivate farmers’ participation in reducing the degradation of forests of the Usambara Mountains in Tanzania. Consideration of such measures under the REDD+ scheme can be important in terms of minimizing OCs. However, depending on the forest products that are considered to be harvested sustainably, such incentives can also involve high management costs and therefore increase the implementation cost of the REDD+ policy. For example, in the studied villages, the firewood collected from the montane forest does not require much management input, but the harvesting of wood from miombo woodlands for charcoal production would require additional management costs if the harvesting were to be kept within sustainable limits.

### REDD+ payments to offset OCs

The results of our analysis showed significant variation in the median compensation required to protect the different vegetation types in the two agro-ecological zones. Several researchers have noted variations in the OCs of emission reduction among tropical locations, depending on ecological and economic conditions (e.g., Grieg-Gran 2008; Strassburg et al. 2009). Further, Fisher et al. (2011) reported a variation (IQR = USD 3.20–5.50 tCO_2_^−1^) in the OCs of avoiding agricultural expansion and charcoal production between 53 districts in eastern Tanzania. In our study, the main difference in the OCs was attributed to variations in biomass (carbon) density between the two vegetation types, and between the miombo woodland in forest reserve and on public land. When labor was valued as cheap, the NPV (USD ha^−1^) of agricultural production was not significantly different between the highland and lowland agro-ecological zones. However, due to pronounced differences in biomass density between the montane forest in the highland and the miombo woodland in the lowland zone, the OC per ton of CO_2_ was significantly different. These findings support those of (Yamamoto and Takeuchi 2012), who have pointed out the significance of variations in carbon density due to vegetation and soil conditions for significant differences in REDD+ compensation estimates. Our estimates of REDD+ payments required to protect the montane forest are similar to estimates reported for humid tropical forests with equivalent carbon densities (e.g., Bellassen and Gitz. 2008; Yamamoto and Takeuchi 2012). However, our estimates for the miombo woodlands are higher than estimates of REDD+ compensation payments reported for other miombo woodlands. For example, Bond et al. (2010) estimated that the regional OC of avoiding the conversion of miombo woodlands to agricultural lands would range from about USD 2.5 tCO_2_^−1^ for Namibia and Mozambique to USD 3.71 tCO_2_^−1^ for Zambia. The differences in compensation estimates are due to variations in assumptions regarding time horizon, discount rate, and carbon density. For example, Bond et al. (2010) used a discount rate of 10 % p.a. and planning period of 30 years. The carbon density considered ranged from 45 to 60 tC ha^−1^, which are between double and triple the carbon density used in our analysis. The net returns to cultivation reported are also significantly lower than the agricultural rents found in our study area.

The differences in estimated carbon payments between the villages within each agro-ecological zone were due to variations in the NPV estimates of crop production, which were attributed to differences in crop type. The highest value was observed for Kunke, mainly due to the relatively high profits from sugarcane and rice cultivation. Variations in compensation values due to differences in farming system (subsistence and cash crop cultivation) have also been noted by Bellassen and Gitz (2008) and by Olsen and Bishop (2009). During our study, we observed that the prices of sugarcane and rice were lower than elsewhere in Tanzania. Based on the information gathered from the growers, we anticipate that the prices of the crops will increase in the near future and may thus increase the profitability of cultivating these crops, which would imply that the potential change in the prices of some crops should be taken into account when designing a REDD+ policy.

Our results suggest that implementing a REDD+ policy may be feasible in the highland agro-ecological zone, where considerable emission reduction could be achieved for a payment of less than the current carbon price (USD 5 tCO_2_^−1^) in the European market (McGrath 2013). Regarding the miombo woodlands, we found that it would be comparatively cheaper to protect the denser miombo woodlands in the forest reserves than the more degraded miombo woodlands on public land. However, the implementation of the REDD+ policy in the lowland zone would generally require higher compensation payments. Further, the analysis was based on assumed carbon payments at stump, which means that the actual carbon payment might be higher than our estimates. Moreover, incorporating implementation costs which according to Fisher et al. (2011) are estimated to be USD 6.50 CO_2_^−1^ on average would further increase the cost of the policy measure.

Our results suggest that implementing the REDD+ scheme may be feasible if the biomass (carbon) density in the studied vegetation is high. However, the relatively dense miombo woodlands and intact montane forest are currently protected as forest reserves, and the present REDD+ schemes do not distinguish official protection status of areas. Nevertheless, implementing REDD+ in the existing protected forests could be an effective and a feasible measure to cut CO_2_ emissions, mainly for three reasons. First, deforestation and degradation is significant in protected areas. For example, between 1975 and 2000, 3.4 % of forest and 28.3 % of miombo woodland were lost from the EAM (Green et al. 2013). Currently, respectively 74 % and 32 % of the remaining forest and woodland in the EAM are within protected areas (Green et al. 2013). Coastal forests in protected areas have been declining too, at a rate of 0.2 % per year since the 1990s (Godoy et al. 2011). Similarly, miombo woodland in the KFR has declined by 6 % between 1964 and 1996 (Luoga et al. 2005). The statistics imply that law enforcement efforts alone have not been sufficient to protect Tanzania’s forests, and therefore economic measures are required to enforce effective protection.

The second reason why implementing REDD+ in the existing protected forests could be an effective and a feasible measure to cut CO_2_ emissions is that the available carbon density in protected forests and woodlands is relatively high compared to forests and woodlands on public land, and the third reason is that the total cost of establishing new forest reserves would be higher than maintaining the existing ones. The median annual actual and necessary management costs in the EAM are estimated to be USD 2.3 ha^−1^ (IQR = USD 1–6 ha^−1^) and USD 8.3 ha^−1^ (IQR = USD 5–17 ha^−1^), respectively (Green et al. 2012), and are much lower than USD 0.1 tCO_2_^−1^. Thus, implementation of the REDD+ policy could be an opportunity to help strengthen established protected areas. Given the fact that about 50 % of Tanzania’s forests and woodlands are within forest reserves, it would be logical to invest in the existing reserves.

### Sensitivity analysis

As with all land valuations, high discount rates imply lower land values, and in the case study also lower compensation levels. Given the level of poverty persisting in rural Tanzania, a discount rate of 10 % per annum may not be entirely unrealistic in estimations of OCs. Most inhabitants in areas of miombo woodland have a very low income and they often prefer immediate consumption, which means they have to apply high discount rates (Bond et al. 2010). In the case of the lowland agro-ecological zone, the increase in discount rate meant that the compensation level in Maseyu was reasonable. Similarly, the implementation of the REDD+ policy could be feasible in Maseyu if the carbon density of the dense miombo is 50 % higher than the density we gathered from secondary sources. On the other hand, a decrease of up to 50 % in the carbon density of the montane forest would not lead to a level where it would become infeasible to invest in reduced emissions, particularly when a positive wage rate is considered. A positive wage rate may be a realistic assumption in areas where rural people can find alternative employment.

## Conclusions

Knowledge of the monetary incentives required to avoid or reduce deforestation and forest degradation in various areas under different settings can help to identify economically feasible locations for the implementation of the REDD+ policy. Our study estimated the financial incentives required to stop the deforestation of both the montane forest in the highland agro-ecological zone and the miombo woodlands in the lowland agro-ecological zone of Morogoro Region, Tanzania. The median compensation required to protect the current carbon stock in the two vegetation types ranged from US$ 1 tCO_2_e^−1^ for the montane forest to US$ 39 tCO_2_e^−1^ for the degraded miombo woodlands. Our analysis revealed that the level of compensation required to avoid the forestland from being converted into cropland depended mainly on the biomass (carbon) density of the vegetation to be protected. Variations in assumptions regarding the OCs of labor and discount rate resulted in significant variations in the OC estimates and hence also the compensation levels between the two agro-ecological zones and between villages within the same agro-ecological zone. In such places as where our study villages were located, where there are few employment opportunities to compensate farmers for the reduction in work as a consequence of implementing REDD+, the level of compensation should be relatively higher. The choice of crop types attributed to both agro-ecological conditions and market access is an important factor in determining the feasibility of the implementation of the policy measure. From our study, we can conclude that given all possible factors that can potentially affect estimates of REDD+ payments, it would be more feasible to implement the policy in the highland zone than in the lowland zone. Depending on the biomass density of the vegetation to be protected, sustainable wood harvesting could be an important incentive under the REDD+ scheme. Moreover, considering factors such as available biomass density, implementation and management costs, and the degree of existing deforestation, the implementation of the REDD+ policy in existing protected areas could be a feasible and effective way to reduce emissions of CO_2_.
